# Infantile Spasms in Pediatric Down Syndrome: Potential Mechanisms Driving Therapeutic Considerations

**DOI:** 10.3390/children11121513

**Published:** 2024-12-13

**Authors:** Carl E. Stafstrom, Li-Rong Shao

**Affiliations:** Division of Pediatric Neurology, Department of Neurology, The Johns Hopkins University School of Medicine, Baltimore, MD 21287, USA; lshao5@jhmi.edu

**Keywords:** Down Syndrome, seizures, infantile spasms, Ts65Dn, TcMAC21, excitation, inhibition, animal models, gamma-aminobutyric acid (GABA), chloride

## Abstract

Infantile spasms are common in Down Syndrome (DS), but the mechanisms by which DS predisposes to this devastating epilepsy syndrome are unclear. In general, neuronal excitability and therefore seizure predisposition results from an imbalance of excitation over inhibition in neurons and neural networks of the brain. Animal models provide clues to mechanisms and thereby provide potential therapeutic approaches. Ts65Dn mice have been the most widely used animal model of DS. In this model, there is evidence for both abnormal cerebral excitation and inhibition: infantile spasms-like clinical and electrographic activity can be elicited by the administration of gamma-aminobutyric acid (GABA)-B receptor agonist, gamma-butyrolactone (GBL), and depolarizing GABA-A responses persist beyond the age of their usual switch to hyperpolarized responses. But despite its widespread use, the Ts65Dn model may be suboptimal because of the absence of numerous genes that are triplicated in human DS and the presence of numerous genes that are not triplicated in human DS. Recently, a transchromosomic mouse artificial chromosome 21 (TcMAC21) mouse model has been developed, which carries a copy of human chromosome 21 and therefore has a genetic composition more similar to human DS. As in Ts65Dn mice, exposure of TcMAC21 mice to GBL results in epileptic spasms, and aberrant excitation has also been demonstrated. This review summarizes excitatory and inhibitory dysfunction in models of DS that may play a role in the generation of seizures and infantile spasms, providing a perspective on past studies and a prelude for future ones. Further elucidation will hopefully lead to rational therapeutic options for DS children with infantile spasms.

## 1. Infantile Epileptic Spasms Syndrome (IESS)—Clinical Features and Animal Models

IESS, formerly called infantile spasms or West syndrome, is an epileptic encephalopathy that begins in children in the middle of the first year of life. Common features of any epilepsy syndrome may include age of onset, seizure semiology, associated signs and symptoms, laboratory findings such as electroencephalogram (EEG) results, genetic predisposition, medication responsiveness, natural history, and prognosis [1] (Table 1). The term epileptic encephalopathy refers to a disorder in which the seizures themselves or the accompanying EEG abnormalities contribute to brain dysfunction and cognitive impairment beyond the underlying pathology [1,2].

IESS is distinct among the epilepsy encephalopathies in several respects. The onset of the unique seizure semiology—flexion, extension, or mixed flexion/extension spasms—occurs at several months of age. IESS can be caused by an enormous spectrum of genetic or acquired etiologies, suggesting that there are some commonalities in the brain’s susceptibility to numerous, diverse brain pathophysiologies at this early stage of brain development. Clinical variability and prognosis depend on the specific etiology of IESS. The congruent EEG patterns, seizure semiology, and underlying neurobiological mechanisms remain poorly understood. Furthermore, the lack of effective treatments and poor cognitive outcomes underlie an urgency to understand the pathophysiology and enhance treatment options for IESS. The most common initial (“first-line”) therapies for IESS are adrenocorticotropic hormone (ACTH) or oral corticosteroids (OCSs); meanwhile, the gamma-aminobutyric acid (GABA) transaminase inhibitor, vigabatrin, seems to be particularly effective in children with IESS due to tuberous sclerosis complex, and it is favored by some clinicians for other etiologies as well [3]. Overall, ACTH or OCSs have better efficacy, but adverse side effects are frequent, and these treatments are not mechanism-specific [4]. Therefore, the search continues for more effective, safer treatments.

Animal models are crucial for elucidating IESS disease mechanisms and thereby revealing potential targets for treatment. While “ideal” criteria for an animal model of IESS have been delineated (i.e., clinical seizure type, age correlation, EEG findings, treatment response, developmental outcome/prognosis), useful mechanistic information can be obtained from an animal model even if all of these criteria are not fulfilled [5]. The array of animal models of IESS mirrors the wide spectrum of IESS etiologies in humans [6,7,8,9,10]. Examples include knockout or poly-alanine expansion of the transcription factor ARX (aristaless-related homeobox gene) [11,12]; a “triple toxin” model with separate chemical agents causing inflammation, oxidative neuronal damage, and serotonin depletion [13]; N-methyl-D-aspartate receptor overactivation caused by prenatal stress [14]; and chronic denervation elicited by tetrodotoxin, a sodium channel blocker [15]. In addition, some newer animal models of infantile spasms have been reported recently, including dysfunction (knockout) of the adenomatous polyposis coli (APC) pathway leading to elevated beta-catenin, causing abnormal brain development [16]; pathogenic microbiome alterations [17]; and mechanistic target of rapamycin (mTOR) pathway abnormalities [18]. Finally, Down Syndrome (DS) animal models of infantile spasms add to the spectrum of clinical-pathophysiological correlations and are discussed in detail below [19]. Of course, some of these seemingly disparate mechanisms could co-occur and add to the synergy of infantile spasms predilection. Together, these models increase the understanding of various aspects of IESS, aiding translational research by identifying rational pathophysiological targets and informing clinical trial design. In DS, many trials based on animal models have been attempted to target cognitive deficits, but there is scant research addressing seizure mechanisms.

## 2. Seizures and Infantile Spasms in Down Syndrome

### 2.1. Clinical Aspects

(DS is the phenotypic expression of triplication of human chromosome 21 (Hsa21) (trisomy 21). The clinical features of DS are well recognized and can involve almost every organ system [20,21]. Neurologically, children with DS display moderate-to-severe intellectual disability, mood disorders, and learning and memory impairments [22]. Furthermore, seizures are common. 

#### 2.1.1. Seizures in DS

**Figure 1:**
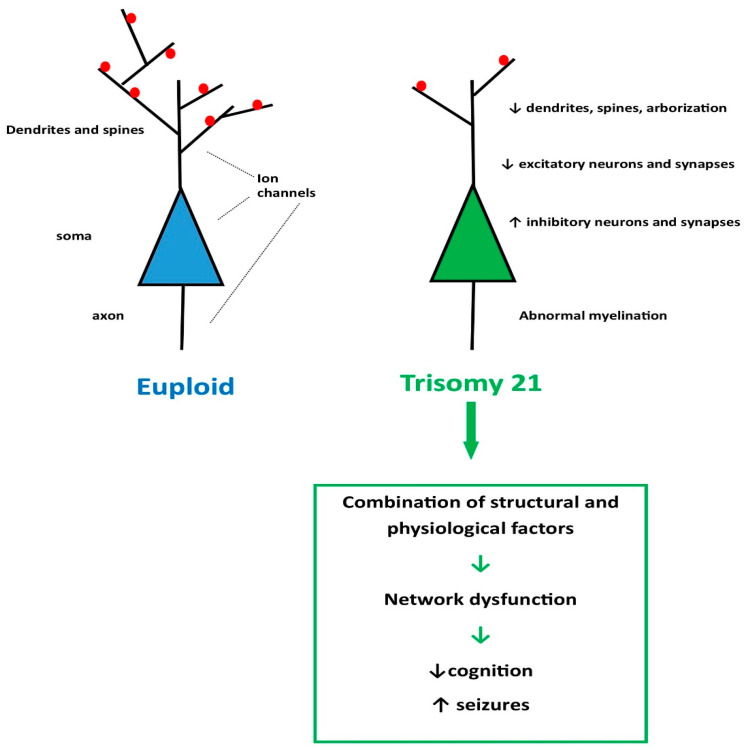
Comparison of hypothetical neuronal structure of a euploid neuron (neocortical or hippocampal principal neuron) and a neuron from a subject with trisomy 21. The euploid neuron embodies a complex dendritic field with numerous dendritic spines (red dots) that largely receive excitatory synaptic input. Ion channels are present throughout the neuron, but the channel subtypes and their distribution vary along the neuronal surface. The soma receives predominantly inhibitory input. The trisomy 21 neuron has a simplified denritic tree with fewer spines. Myelination patterns are also aberrant. This combination of physiological and structural differences and their network connections underlie cognitive dysfunction and increased seizure susceptibility in Down Syndrome. See text and Table 2 for details. Green arrows refer to the sequential development of dysfunction in cognition and seizures; black arrows refer to the direction of change (decrease or increase).

Seizures affect 1–13% of individuals with DS over their lifetime, as reported in numerous clinical surveys [23,24,25,26]. Seizure types include generalized tonic-clonic, focal aware or unaware, myoclonic, atypical absence, atonic, and infantile/epileptic spasms (see Section 2.1.2). The increased seizure predisposition in DS is likely a consequence of either of two broad pathologies: (1) abnormalities of brain development and function related to trisomy of chromosome 21 (Figure 1, Table 2); or (2) consequences of medical complications experienced by children with DS, such as increased susceptibility to infections and leukemia, immune dysregulation, congenital heart disease, and other comorbidities [25,27]. Later in life, when individuals with DS mature into their thirties, myoclonic seizures often emerge (known as LOMEDS or late-onset myoclonic epilepsy in DS), around the same time as Alzheimer-like dementia begins in these individuals. It is possible that the accumulation of ß-amyloid causes cortical irritability and increased susceptibility to myoclonic seizures. This critically important topic is discussed in recent reviews [28]. Here, we focus on early life seizures in DS. 

Because seizures, especially epileptic encephalopathies such as IESS, are often associated with long-term learning and cognitive impairments, it is particularly important to effectively treat seizures in children with DS, who already harbor cognitive challenges and intellectual disability, lest the seizures induce even greater impairments later in life. The main question and challenge is to determine whether seizures are the inevitable byproduct of abnormal brain development in DS or whether seizures represent a target worth pursuing to preserve cognition and provide neuroprotection.

#### 2.1.2. Infantile Spasms in DS

IESS is the most common epilepsy syndrome in children with DS, with clinical features and epidemiology similar to other IESS etiologies. The literature is quite consistent that between 0.6 and 13% of individuals with DS develop IESS (prevalence 6–46%), representing a 100-fold increase over the general population [26,27,29,30,31,32]. Reasons for this increased IESS predisposition are unknown; it might be speculated that one or more genes triplicated in trisomy 21 could be responsible, at least in part, but the complex genotype-phenotype correlations remain unresolved, and epigenetic factors in DS may also contribute [33,34,35,36]. The genetic basis of phenotypic manifestations in DS is still actively being studied and debated.

As noted above, IESS arises from a huge diversity of etiologies, from genetic mutations to acquired disorders such as infections and other brain injuries, all resulting in the expression of a similar clinical syndrome. To generate infantile spasms, the excitation/inhibition ratio within neurons or the neural circuitry in infants with DS must become imbalanced, with the enhanced excitation predisposing to IESS in DS as well as any other IESS etiology [37,38].

### 2.2. Brain Structure and Function in Down Syndrome

Macroscopically, brains of individuals with DS have smaller volume, and the cerebellum is especially undergrown. Microscopically, numerous changes have been characterized, including dendrite and dendritic spine dysgenesis with decreased arborization, abnormal cortical lamination, and smaller hippocampal dentate gyrus (Figure 1 and Table 2). In DS, there is reported to be a relative increase in inhibitory interneurons over excitatory principal neurons [37]. Various factors that increase excitation and/or decrease inhibition are considered below with regard to animal models of DS. These include ion channels, synapse type and function, dendritic structure, and other physiological and structural factors. Any of these factors could engender abnormal firing patterns that underlie cognitive and developmental deficits as well as aberrant excitability and seizures. Overall, the structural anomalies of DS brain have been colloquially summarized as “fewer neurons, abnormally connected” [39].

**Table 2:** Brain developmental anomalies in human Down Syndrome with selected references.

Decreased neurogenesis	Haydar and Reeves, 2012 [40]
Dendritic atrophy (decreased connectivity)	Becker et al., 1986 [41] Takashima et al., 1981 [42]
Dendritic spine dysgenesis and decreased arborization (decreased connectivity)	Bartesaghi, 2023 [39]
Delayed and disorganized cortical lamination	Goldman and Hyman, 1994 [43]
Smaller, hypocellular hippocampal dentate gyrus	Sylvester, 1983 [44] Guidi et al., 2008 [45]
Hypoplastic cerebellum	Aylward et al., 1997 [46] Guidi et al., 2011 [47]
Cortical pyramidal neurons: prenatal—smaller dendritic arbors and fewer synapses; postnatal—pyramidal neuron dysgenesis, dendritic and synaptic abnormalities; decreased total neurons in cortex and hippocampus	Marin-Padilla, 1976 [48] Suetsugu and Mehrein, 1980 [49] Vuksić et al., 2002 [50]

## 3. Animal Models of Down Syndrome

**Figure 2:**
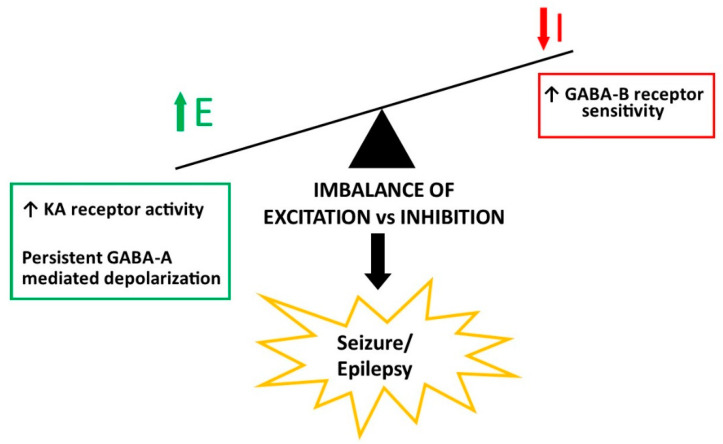
Schematic illustrating some potential seizure mechanisms in Down Syndrome. Any factor that increases excitation, decreases inhibition, both can lead to neuron or network hyperexcitability and seizures. The mechanisms noted in the boxes represent possible contributors to seizures/epilepsy in Down Syndrome, with triplicated genes for those factors being located on chromosome 21 (KA receptors) or shown in animal models (GABA-A and GABA-B receptors). E, excitation; I, inhibition; GABA, gamma-aminobutyric acid; KA, kainic acid. The green arrow refers to increased excitation, and the red arrow refers to decreased inhibition.

More than 20 animal models of DS have been created, some of which hold considerable promise for understanding this neurodevelopmental disease and identifying potential treatment options [33,35,51]. While no animal model can replicate all aspects of a human disease, certain animal models of DS have provided important preliminary information about the role of triplicated genes and gene interactions on disease manifestations and progression. While spontaneous seizures have not been observed in any DS mouse model to date, important information about seizure threshold, predisposition, and propagation can be obtained from exposure to agents that induce seizures. Indeed, in some DS animal models, seizures—and even spasms—can be induced pharmacologically. Two such models are highlighted here, the Ts65Dn mouse and the transchromosomic mouse artificial chromosome 21 (TcMAC21) mouse. In each model, seizure generation can be conceptualized as a disruption in the brain’s usual balance between excitation and inhibition, with neuronal or network excitability taking precedence over inhibition (Figure 2) [38,52,53]. Contributors to excitation/inhibition balance include the wide array of ion channels, neurotransmitters and their receptors, and neuronal connectivity, alteration of which can lead to network hyperexcitability. While a multitude of such mechanistic alterations may be present in DS, here we focus on a couple of the more likely ones for which experimental data are available or in which the genetic defect is likely to be contributory. It should be recognized that, in addition to conventional ion channel and synapse dysfunction, emerging alternative mechanisms can increase neuronal hyperexcitability, including epigenetic, environmental, and immunological mechanisms; at present, little is known about the relationship of these factors to seizures in DS, either clinically or experimentally.

### 3.1. Ts65Dn

The Ts65Dn mouse is the most extensively studied animal model of DS, with publications spanning more than three decades [54]. This trisomic mouse carries an extra copy of the distal segment of mouse chromosome 16, which is analogous to the long arm of Hsa21 (Table 3).

Ts65Dn replicates the hippocampus-based cognitive deficits of human DS, including impaired synaptic plasticity and learning and memory deficits [55]. Spontaneous seizures have not been reported in Ts65Dn mice (nor in any other DS model). It is possible that Ts65Dn mice are particularly susceptible to audiogenic seizures, but only preliminary data are available [56]. The extensive literature on Ts65Dn mice concludes that altered gene dosage perturbs development, affects brain morphogenesis and function, and engenders decreased synaptic plasticity combined with excessive inhibitory interneurons [57], leading to the characteristic cognitive profile of DS. Abnormalities of dendritic development have been documented in hippocampal granule neurons and neocortical neurons of Ts65Dn mice [58,59]. Myelination abnormalities may also contribute to altered circuit function [60].

Yet, caution is required because it has become increasingly recognized that Ts65Dn is an incomplete model of DS. While this model has provided some valuable insights into the pathophysiology of DS, including cognitive, learning, and memory dysfunction [39,61,62], it has evident limitations: (1) the Ts65Dn mouse is trisomic for only 60% of conserved protein-coding genes homologous to Hsa21 [40,54]; and (2) the Ts65Dn mouse harbors about 30 trisomic protein coding genes whose human homologs are not found on Hsa21 [63]. Furthermore, while certain treatments rescued the cognitive deficits in Ts65Dn mice, attempts to replicate this situation in humans with DS proved ineffective [64]. Thus, despite its widespread use, questions have arisen as to whether the Ts65Dn model is applicable to human DS. Therefore, the results summarized here must be interpreted in the context of the limitations of the Ts65Dn model [33,63]. Newer models, such as the Ts66Yah mouse model, are derived from the Ts65Dn lineage but do not harbor the duplicated mouse chromosome 17 centromeric region; but thus far, there are no data on neuronal excitability or seizure predisposition [63,65,66]. In considering any model system, the reader is reminded that human conditions occur only in human beings. There is no perfect molecular, animal, or cell model of any human disease—this is inherent in the meaning of “model”.

#### 3.1.1. GABA Receptors and Inhibition in Normal Brain

GABA is the major inhibitory neurotransmitter in the brain. GABA exerts its inhibitory action by binding to one of two types of GABA receptors, GABA-A and GABA-B. GABA-A receptors mediate fast inhibitory neurotransmission via ligand-mediated binding of GABA to GABA-A receptors, which opens a chloride (Cl^−^) channel; influx of Cl^−^ hyperpolarizes the neuron, keeping the membrane potential from reaching the firing threshold. GABA-B receptors are G-protein-coupled receptors that activate an inward rectifier-type potassium channel (GIRK); outward K^+^ flux through this channel hyperpolarizes the neuron. GABA-B receptors are also present presynaptically where their activation prevents neurotransmitter release by acting via voltage-gated calcium channels. A final mechanism by which GABA-B receptors facilitate inhibition is by mitigating the effects of concurrent excitatory postsynaptic potentials by “shunting” the excitatory current. Perhaps paradoxically, as illustrated here, there are several mechanisms by which increased inhibition in neural circuits can actually facilitate excitation and synchronize neuronal firing, based on specific abnormalities in the function and connectivity of inhibitory interneurons, to cite just one example [67,68,69].

##### GABA-A Receptor Dysfunction

The pathological basis of Ts65Dn-related neuronal dysfunction has been attributed to the presence of excessive forebrain (GABAergic) interneurons, leading to enhanced inhibition that alters synaptic plasticity and cognition [37,39,52,57].

GABA-A receptor antagonists reduce GABAergic signaling and, to some extent, rescue some of the deficits. However, this reasoning raises the question of why excessive inhibition would present as increased excitability and seizures, while the opposite effect might be anticipated—that too much inhibition would *lessen* the chance of seizures. Deidda and colleagues addressed this conundrum and showed that in Ts65Dn mice, excess of receptor type-A GABAergic signaling causes unbridled excitation because of developmental dysregulation of Cl^−^ homeostasis, resulting in prolonged and excessive GABA-induced depolarization rather than GABA’s typical hyperpolarizing effect [70]. In the developing brain, GABA-A depolarizing responses are essential for certain trophic functions and normal synaptogenesis; the polarity (depolarizing or hyperpolarizing) of GABA responses at a particular age is due to differential developmental expression of Cl^−^ transporters [71]. Early in development (until approximately the late premature stage), predominant expression of the sodium-potassium chloride co-transporter (NKCC1) creates a higher Cl^−^ concentration intracellularly than extracellularly; therefore, GABA-A receptor activation causes depolarization. Within a few weeks after birth, NKCC1 expression diminishes and a Cl^−^ exporter, potassium-chloride co-transporter 2 (KCC2), is preferentially expressed [72]. KCC2 keeps the extracellular Cl^−^ concentration higher than the intracellular Cl^−^ concentration so that GABA-A receptor activation results in hyperpolarization. However, if depolarizing GABA responses persist beyond the expected developmental window [73], abnormal neuronal function and even seizures can result. In this regard, treatment of the young mice with bumetanide, an inhibitor of NKCC1, or with artificial microRNAs (amiRs) rescues long-term potentiation and memory deficits and restores GABA-A receptor signaling in Ts65Dn mice [70,74] and might represent a potential rational therapy for seizures as well. Moreover, treatment of Ts65Dn mice with the benzodiazepine diazepam (a GABA-A-positive allosteric modulator) prior to picrotoxin (GABA-A receptor antagonist)-induced seizures limited seizure susceptibility and severity [75].

##### GABA-B Receptor Dysfunction

Extensive research has implicated GABA-B receptors in the cognitive dysfunction of Ts65Dn mice, in which pharmacological or genetic reduction of GABA-B receptor function rescues cognitive impairments [76]. However, this observation would not explain increased seizure susceptibility. Cortez and colleagues found that GABA-B overactivation causes hyperexcitability and epileptic spasms in Ts65Dn mice [19]. One gene that is overexpressed in the Ts65Dn mouse is *kcnj6*, located on mouse chromosome 16 and on the long arm of Hsa21. It codes for the G-protein-coupled inward-rectifying potassium (GIRK2) channel protein. *kcnj6* overexpression leads to enhanced GABA-B receptor-mediated GIRK currents, which are outward K^+^ currents that lead to extracellular K^+^ accumulation and further depolarization [77]. The investigators showed that Ts65Dn mice are extremely sensitive to the induction of an infantile spasms-like phenotype induced by GABA-B receptor agonists (gamma-butyrolactone (GBL)), whereas GIRK2 knockout mice are resistant to spasms induction. However, *kcnj6/GIRK2* single-gene triploid mice failed to show susceptibility to the infantile spasms phenotype. These data suggest that overexpression of GIRK2 in the brain is necessary but not sufficient to confer susceptibility to GABA-B receptor agonist-induced infantile spasms in the Ts65Dn model of DS [78]. The authors concluded that it is likely that GIRK2 works in concert with another factor or factors that are altered in the Ts65Dn brain to produce the GABA-B agonist-induced infantile spasms phenotype.

#### 3.1.2. Excitation Abnormalities

Less has been studied about the excitatory side of the E/I imbalance in DS. One report with Ts65Dn mice found that neocortical layer IV neurons were less excitable than neurons from euploid mice due to increased membrane capacitance and decreased membrane resistance [79]. However, an extensive compilation of studies concluded that there are no consistent major abnormalities in passive membrane properties (e.g., resting membrane potential, input resistance, action potential threshold) in Ts65Dn neurons [39], so passive properties are not likely to be the primary culprit [80]. The role of the kainic acid (kainate, KA) subtype of glutamate receptors in DS is unclear but requires additional study because the gene for KA receptors (KARs) resides on chromosome 21. DYRK1A, a gene located on Hsa21 and involved in glutamatergic neuron development, is overexpressed in Ts65Dn mice and has been associated with learning and memory deficits, but does not affect seizure susceptibility [81].

### 3.2. TcMAC21

Recently, Reeves and colleagues developed a mouse model of DS called TcMAC21 that is genetically engineered to carry a copy of Hsa21 [82]. This mouse is not mosaic and is considered to represent the animal model with the most realistic similarity to the human condition to date, carrying 93% of Hsa21 protein coding genes (compared with 60% in Ts65n). TcMC21 mice recapitulate the DS phenotype with respect to several body systems including cardiac, skeletal, and neurological. Neurological features include structural (smaller brain volume including cerebellum; ventriculomegaly) and functional (diminished synaptic plasticity: decreased long-term potentiation and learning/memory deficits) abnormalities of the brain. Importantly, while TcMAC21 mice demonstrate structural and functional abnormalities as also seen in Ts65Dn mice, the deficits in TcMAC21 mice are milder, probably related to Ts65Dn mice harboring many gene variants not present in TcMAC21 mice or in humans with DS.

We recently examined seizure and epileptic spasms susceptibility in TcMAC21 mice [83]. Following exposure to the GABA-B receptor agonist GBL, both TcMAC21 mice and their euploid littermates developed spasm-like activity, but the prevalence of spasms was far greater in TcMAC21 mice—about 85% of TcMAC21 mice developed spasms, while only 25% of euploid littermates did so. GBL elicits rhythmic sharp waves and spikes on EEG, with spasms occurring only during sharp-wave bursts. Search for reasons why neuronal excitability is increased in TcMAC21 mice revealed no differences in passive membrane properties compared with euploid controls (similar to Ts65Dn neurons as described above), but TcMAC21 mice did have significantly larger excitatory postsynaptic currents (but not inhibitory postsynaptic currents) in layer V neocortical neurons, leading to an increased E/I ratio [83]. Therefore, increased neocortical excitation in this DS genetic model could enhance the susceptibility to infantile spasms; mechanisms for this increased excitability are currently being investigated. At least three genes triplicated in TcMAC21 mice could play a role (Figure 2). First, KA-type glutamate receptors (*KAR1* gene): TcMAC21 neurons are more sensitive to KA application than are euploid neurons (Shao, L.-R. (Johns Hopkins University, Baltimore, MD, USA), unpublished observations). Second, although GBL elicits epileptic spasms more readily in TcMAC21 mice, neurons from TcMAC21 and euploid mice respond similarly on a cellular basis to GBL, suggesting multiple roles for GABA-B receptors in this model. Finally, the synaptic vesicle trafficking protein, synaptojanin, encoded by *SYNJ1*, has been hypothesized to play a role in regulation of neuronal excitation. Synaptojanin is a lipid phosphatase involved in vesicle uncoating and recycling [84]. Although not yet studied in DS models, *SYNJ1* variants have been reported in epileptic encephalopathy, so they warrant further study [85]. In efforts to elucidate the excitability and seizure mechanisms in TcMAC21, it is acknowledged that the molecular genetic contributions from these and other (non-chromosome 21) genes could also be involved. To develop optimal treatments based on mechanism, all of these avenues should be explored.

## 4. Is There a Preferred Standard Treatment for Infantile Spasms in Children with Down Syndrome?

Despite infantile spasms being common in DS, the number of patients with both conditions is limited. This reality has hindered large cohort studies to determine the best treatment option, and most reports for such recommendations are anecdotal. In infantile spasms overall, many medications have been tried, with variability in study design, dose, treatment duration, and other factors limiting conclusions [86]. Standard or “first-line” medications have included adrenocorticotropic hormone (ACTH), oral corticosteroids (OCSs), and vigabatrin. (Collectively, ACTH and OCSs are referred to as “hormonal” therapies.) Of those agents, most of the evidence, though anecdotal or based on small series, favors hormonal therapies over vigabatrin [31,87,88,89,90,91]. Several nonstandard medications, including but not limited to topiramate, benzodiazepines, valproic acid, zonisamide, etc., are not preferred and have minimal evidence for effectiveness. Emerging data using larger, multi-center cohorts such as the Pediatric Epilepsy Research Consortium, also support hormonal therapy (Chen, H. (UCSF, San Francisco, CA, USA), unpublished observations). Early diagnosis and treatment will likely exert a favorable long-term effect, as DS children with infantile spasms have poorer neurodevelopmental and epileptic outcomes than DS patients without IESS [92,93].

As for treatment of IESS overall, a personalized, precision medicine approach would be optimal, or at least a strategy that addresses the pathophysiological mechanism(s) leading to spasms. Of note, the hormonal therapies (ACTH and OCS) are relatively nonspecific mechanistically, while the only medication among the standard therapies with a known mechanism of action is vigabatrin, which restores GABAergic function by irreversibly inhibiting GABA transaminase. Yet, vigabatrin’s effectiveness for spasms in DS does not appear to be robust. Other GABAergic agents such as bumetanide, a diuretic that inhibits the chloride co-transporter NKCC1 and enhances the hyperpolarizing action of GABA, has not yet undergone clinical trials for IESS in DS, and its potential role in neonatal seizures is controversial [94,95]. However, bumetanide (already discussed above) and other agents with specific pathophysiological correlations do hold promise for seizure treatment in DS. One example would be potentiation of KCC2 function, which has been effective in some epilepsy and cognition models [96,97]. In hippocampal neurons, there was similar KCC2 expression in Ts65Dn mice, euploid controls, and humans with DS, but the authors state that KCC2 expression variability was high, and a role for KCC2 could not be excluded [70]. 

Other agents for refractory epileptic spasms include cannabidiol (CBD) and the ketogenic diet (KD). In a small case series, seven DS children with IESS unresponsive to first-line agents were trialed with CBD oil; five of the seven children achieved more than 50% spasms reduction [98]. Data on the KD are very sparse. One case report describes complete abatement of spasms and cognitive improvement in a DS child whose spasms were unresponsive to hormonal therapies and vigabatrin, but details are not provided [99]. This metabolic approach to decreasing neuronal excitability is appealing but requires considerable additional verification [100]. Gene therapies have also been proposed as potentially beneficial, but there is no imminent application of such techniques to seizure management in DS.

## 5. Conclusions, Research Gaps, and Treatment Challenges and Opportunities

Treatment of seizures, and infantile/epileptic spasms in particular, is a critical unresolved research and clinical problem. Extant and future animal models of DS will contribute to mechanistic understanding and open therapeutic avenues. Potential therapeutic targets, based on rational molecular pathologies, include GABA receptors (both A and B types) and glutamate receptors (especially KA receptors); additional possibilities may well emerge as basic science and clinical research progress. 

Many opportunities exist for further study that could elucidate not only seizure predisposition but also trisomy 21-mediated dysfunction of other organ systems in DS:

- Epigenetic, environmental, and immunological targets in DS may become available and are already being addressed [34]. For example, in boys (but not girls) with DS, DNA hypomethylation markers were greater in those with congenital heart defects than those without, suggesting sex- and disease-specific epigenetic changes [101]. In the brain, Ts65Dn mice display DNA methylation markers suggesting a more advanced hippocampal epigenetic age than euploid mice [102], similar to human DS [103]. It is well established that individuals with DS have chronic hyperactivation of interferon signaling (interferonopathy) with immune system dysregulation, and an emerging literature is exploring the underlying immunopathologic mechanisms as well as treatment potential of JAK/STAT (Janus kinase/signal transducer and activator of transcription) inhibition in restoring interferon activity in DS individuals [104]. Of note, knockdown of the JAK/STAT pathway has been shown to reduce seizures and improve cognition in rodent models of temporal lobe epilepsy [105].
- Model systems: As in experimental efforts to elucidate mechanisms of seizures and epileptogenesis in general [106], model systems have expanded beyond rodents to include zebrafish, Drosophila, brain slices and organotypic slice cultures, human-derived induced pluripotent stem cells (iPSCs), and others [33,35,51,67]. Each of these alternative model systems can be used to ask specific questions about neuronal excitability and its control, recognizing that such reductionism addresses molecular mechanisms more than circuit dysfunction. Presently, there is minimal information using these systems for seizures in DS, but one could envision both electrophysiological and genetic dissection of the effects of trisomic genes on membrane excitability using such simpler systems. While it is unlikely that robust research will emerge using either higher-order mammals or nonhuman primates, a transchomosomic rat model of DS (called TcHSA21rat) has been developed recently, genetically similar to the mouse TcMAC21 [107]. The larger size of rats expands the types of cognitive and physiological testing that can be performed; as of yet, there have been no studies of seizure susceptibility using this rat.
- Risk factors for the development of seizures or IESS in DS: To date, no specific associations have been identified as risk factors for seizures or IESS among individuals with DS.
- Available treatments and prognosis of epilepsy and cognition: At present, for children with DS, infantile spasms appear to be more responsive to hormonal therapies than to vigabatrin. An additional critical and presently unresolved issue is whether seizures (or spasms) in children with DS further exacerbate underlying cognitive and functional status or cause any structural damage; no previous studies have investigated this question, but it is pivotal in deciding how aggressively seizures in DS should be treated. Finally, the lack of observed or recorded spontaneous seizures in any DS animal model remains an intrinsic concern for model validity (Table 3), although it is possible that spontaneous seizures do occur but have been missed given the relatively low prevalence and random nature of seizure occurrence, which will require monitoring a large number of animals over a prolonged period to make a definitive conclusion.

## Figures and Tables

**Table 1:** Infantile epileptic spasms syndrome.

Age of onset	Mid-first year of life (~4–10 months)
Seizure semiology	Flexion or extension spasms
Etiologies	Numerous, spanning genetic and acquired causes
Associated signs and symptoms	Cognitive-developmental plateau or regression
EEG features	Interictal: hypsarrhythmia Ictal: electrodecrement
Treatment responsiveness	Corticosteroids (prednisolone), adrenocorticotrophic hormone (ACTH), vigabatrin
Prognosis	Poor: subsequent cognitive impairments and predisposition to other seizure types, dependent on specific etiology

**Table 3:** Animal models of seizures in Down Syndrome.

Altered Function	Ts65Dn Mice	TcMAC21 Mice
DS manifestations and consequences	DS-like outcomes in cognition and plasticity	Similar to Ts65Dn but milder abnormalities
Cell cycle duration	Longer in VZ, SVZ	Unknown
VZ output	Decreased	Unknown
Brain size	Smaller cerebellum	Smaller cerebellum
Neuron numbers	Fewer E neurons, more I neurons	Unknown
Spontaneous seizures	None	None
Spasms-like activity	Increased in response to GBL	Increased in response to GBL

Abbreviations: DS, Down Syndrome; VZ, ventricular zone; E, excitatory; I, inhibitory; GBL, gamma-butyrolactone.
